# Physical activity among hospitalized older adults – an observational study

**DOI:** 10.1186/s12877-017-0499-z

**Published:** 2017-05-16

**Authors:** Sigurd Evensen, Olav Sletvold, Stian Lydersen, Kristin Taraldsen

**Affiliations:** 1 Department of Geriatrics, St Olavs University Hospital, Trondheim, Norway; 2 Department of Neuromedicine and Movement Science, NTNU, Faculty of Medicine and Health Sciences, Trondheim, Norway; 3 Regional Centre for Child and Youth Mental Health and Child Welfare, Department of Mental Health, Faculty of Medicine and Health Sciences, NTNU, Norwegian University of Science and Technology (NTNU), Trondheim, Norway

**Keywords:** Activity monitoring, Accelerometer, Physical activity, Hospitalization, Geriatrics

## Abstract

**Background:**

Low level of physical activity is common among hospitalized older adults and is associated with worse prognosis. The aim of this paper is to describe the pattern and level of physical activity in a group of hospitalized older adults and to identify factors associated with physical activity.

**Methods:**

We measured physical activity on day three after admission using accelerometer based activity monitors and time in upright position as outcome measure. We collected data of physical function (Short Physical Performance Battery, SPPB. 0–12), cognitive function (Mini Mental Status Examination, MMSE, 0–30 and diagnosis of cognitive impairment at discharge, yes/no), personal Activities of Daily Living (p-ADL, Barthel Index, BI, 0–20) and burden of disease (Cumulative Illness Rating Scale, CIRS, 0–56). We analyzed data using univariable and multivariable linear regression models, with time in upright position as dependent variable.

**Results:**

We recorded physical activity in a consecutive sample of thirty-eight geriatric patients. Their (mean age 82.9 years, SD 6.3) mean time in upright position one day early after admission was 117.1 min (SD 90.1, *n* = 38). Mean SPPB score was 4.3 (SD 3.3, *n* = 34). Mean MMSE score was 19.3 (SD 5.3, *n* = 30), 73% had a diagnosis of cognitive impairment (*n* = 38). Mean BI score was 16.4 (SD 4.4, *n* = 36). Mean CIRS score was 17.0 (SD 4.2, *n* = 38). There was a significant association between SPPB score and time in upright position (*p* = 0.048): For each one unit increase in SPPB, the expected increase in upright time was 11.7 min. There was no significant association between age (*p* = 0.608), diagnosis of cognitive impairment (*p* = 0.794), p-ADL status (*p* = 0.127), CIRS score (*p* = 0.218) and time in upright position. The overall model fit was R^2^ 0.431.

**Conclusion:**

Participants’ mean time in upright position one day early after admission was almost two hours, indicating a high level of physical activity compared to results from similar studies. Physical function was the only variable significantly associated with physical activity indicating that SPPB could be a useful screening tool and that mobilization regimes should be delivered routinely for patients with reduced physical function.

## Background

Ability to walk or use a wheelchair is important to be able to live an independent life. About 30% of older adults have difficulties walking a short distance outdoors, and for those with limitations in Activities of Daily Living (ADL) 30% have difficulties crossing a small room [1]. Hospitalization is a risk factor for further loss of ambulatory ability [2, 3]. In addition to being hospitalized because of a present disease, factors like inappropriate medication, prolonged fasting and unnecessary immobilization can also contribute to loss of ambulatory ability [4]. Increasing numbers of studies have evaluated physical activity among older patients during hospital stay [4–8]. Nurse observations among 118 medical patients with mean age 74.4 years indicate a median value of only 5.5 min of ambulating in the hallways at daytime [5]. Studies using accelerometer-based technology also find low levels of physical activity: Brown and colleagues reported that 47 male medical patients with mean age 73.9 years spent 3.7% of their total time standing and/or walking [4]. Pedersen and colleagues reported that 48 ambulatory patients with mean age of 84.7 years spent 4.6% (66 min) of their total time standing and/or walking [6]. Villumsen and colleagues reported that 100 geriatric patients with mean age 84 years spent 5.8% (83 min) of their total time standing and/or walking [7]. Reporting step count, Fisher and colleagues found 739.7 steps per day on average in a population of 239 hospitalized patients with a mean age of 76.6 years [8]. Ostir and colleagues investigated activity in 224 patients with mean age of 76.1 years admitted to an Acute Care for Elders hospital finding that the participants were active 80 min the first 24 h of hospital stay [9]. In summary, these studies on hospitalized older patients find a low level of physical activity.

This low level of physical activity is of concern because physical activity during hospitalization has prognostic impact. Ostir and colleagues report an association between increased physical activity and reduced two-year mortality [9]. Low level of physical activity probably contributes to loss of walking ability which is reported to occur in 17–65% of hospitalized older adults [2, 3]. In view of the prognostic impact, factors associated with physical activity during hospitalization could help guide healthcare. Advanced age, presence of delirium, mobility impairment before admission, a history of falls [8] and cognitive impairment diagnosed at admission [6] are associated with lower level of physical activity during hospitalization, while use of walking aids and more than four comorbid conditions [2] are reported as risk factors for loss of walking ability. Most studies describing physical activity and factors associated with physical activity in hospitalized older adults have important limitations. Some studies have excluded [2, 9] or partly excluded [4, 8] patients with cognitive impairment, others have excluded patients with important geriatric conditions like musculoskeletal disorders, neurological disorders and injuries [9]. Furthermore, reports using direct clinical observation could underestimate the total level of physical activity [5].

In a large randomized controlled trial (RCT) our research group recently compared physical activity in hip fracture patients treated in a conventional orthopedic ward versus hip fracture patients treated in a geriatric hip unit and found higher levels of physical activity in the geriatric hip unit [10, 11]. Because such intervention studies tend to increase the level of physical activity above normal level we therefore wanted to explore physical activity in a geriatric care pathway during hospital treatment of geriatric patients with acute medical conditions, separated from a specific interventional program or trial. The aim of this study is thus two-folded: 1) to describe a population of hospitalized geriatric patients and their level of physical activity measured by activity monitors, 2) to explore if physical function, age, diagnosis of cognitive impairment, function of personal Activities of Daily Living (p-ADL) and comorbidity are associated with physical activity during hospitalization.

## Methods

### Study design and ethics

This is an observational study conducted in the geriatric ward at St Olavs Hospital, Trondheim University Hospital, Norway, between December 16 2013 and March 3 2014. During the study, all patients admitted to the geriatric ward, were eligible for inclusion. The only exclusion criterion was inability of getting informed consent from the patient or a proxy in case of significant cognitive impairment. The Regional Committee for Medical and Health Research Ethics of Mid-Norway approved the study (REK 2013/1357–1).

### Setting and participants

Department of Geriatrics, St Olav University Hospital in Trondheim, Norway, consists of a 15-bed medical geriatric ward, an outpatient clinic, and liaison services to the rest of the hospital and catchment area. About 90 % of the patients are admitted acutely, of which most are frail with extensive co-morbidity and presenting problems like acute or subacute functional decline, where a mix of conditions like impaired cognition (delirium/dementia), immobility, imbalance, incontinence, inappropriate drug-lists, malnutrition/weight loss are prevalent, often triggered by infections, gastroenterological, cardio- and cerebrovascular diseases. This is the same ward as used in the hip-fracture trial [10, 11].

The patients receive comprehensive geriatric care, a well-established and evidence-based approach for treatment and care of older patients [12], in concert with treatment of acute diseases. A geriatric team assesses all patients; the team consists of physicians, nurses, physiotherapists and occupational therapists where all members have a special focus on identifying and mobilizing frail older patients at risk of immobilization. In selected cases, elements of acute rehabilitation within a 5–7-day perspective are added, although specific rehabilitation usually takes place in nursing homes or rehabilitation facilities. The aim is generally to discharge patients to their own home. A substantial number of patients will need short-term nursing home stays.

### Measurements

To measure physical activity we used body-worn, single-axis accelerometer-based devices (35 × 53 × 7 mm, 15 g, activPAL, PAL Technologies Ltd., Glasgow, United Kingdom) attached by a waterproof tape to the midpoint of anterior right thigh. Use of activPAL is a valid method for quantifying physical activity in older people with impaired function, including hospitalized geriatric patients. Due to slow gait speed, step count is not accurately measured by this monitor in this population [13]. We chose day three after admission for activity analysis because most participants completed 24-h activity monitoring this day (*n* = 28, mean 3.2 days after admission, SD 0.9), but included recordings from other days early after admission if day three was missing. We used only complete 24-h activity recordings to describe physical activity. We used time spent in an upright (standing and walking) position as outcome of physical activity. We registered number of upright events, lengths of upright events (minutes), maximum length of upright events (minutes), upright event variability (Interquartile range, IQR, of the length of the upright events) and time spent in upright position during night (00–06), morning (06–12), afternoon (12–18) and evening (18–24) to better characterize the participants’ pattern of physical activity.

We derived information about duration of upright events from the manufacturer’s Excel spreadsheets from software version 7.3.32 (activPAL, PAL Technologies Ltd.) and a custom made MATLAB (MATLAB version 7.1. The MathWorks Inc., Natick, MA, 2005) program to write an Excel spreadsheet (Office Excel version 11.0, Windows XP Professional, Microsoft, 2003) with outcome values for all participants. The minimum length of an upright event for the sample was 9.9 s. To evaluate physical function we used the Short Physical Performance Battery (SPPB), assessing standing balance, 4-m walking, and ability to rise from a chair, providing a total score from 0 to 12, where 12 is best score and suggest better mobility [14]. From the 4-m walking test we calculated preferred gait speed (m/s). To evaluate cognitive status we collected diagnosis of cognitive impairment at discharge (yes/no) and scored Mini Mental Status Evaluation (MMSE) ranging from 0 to 30 where 30 is best possible score [15]. To evaluate function in personal Activities of Daily Living (p-ADL) we used Barthel Index (BI) ranging from 0 to 20 where 20 is best score indicating independency in p-ADL [16]. To evaluate the level of chronic disease we used the Cumulative Illness Rating Scale (CIRS) ranging from 0 to 56 where zero indicates no health problems and the hypothetical score of 56 would indicate severe failure in 14 different systems [17]. To evaluate the burden of acute disease and quantify the level of deviating vital signs we scored a modified APACHE II treating missing values as normal. APACHE II ranges from 0 to 71, where increasing score corresponds to increasing mortality rate [18].

### Data collection

Ward nurses included the participants all days as soon as possible after admission. A trained physiotherapist programmed the activity monitors, and the physiotherapist or ward nurses attached the activity monitors immediately after inclusion. The participants wore the activity monitor continuously (day and night, also during showering) until the day of discharge when a nurse removed the device. The physiotherapist downloaded activity-monitoring data using the activPAL™ software v7.3.32. By visual inspection, the physiotherapist checked all data in the software output for potential errors such as non-wear time (long periods without any change in positions against nurse reports) and incorrect attachment. Physiotherapists performed the SPPB the first weekday after admission, where participants who was not able to perform any parts of the SPPB got the score zero and those not evaluated was missing in the analysis. An occupational therapist completed the MMSE when a doctor considered the patient free of delirium or acute somatic disease (*n* = 30). A medical doctor (MD) collected diagnoses of cognitive impairment from case records and discharge reports retrospectively, as well as information on use of home services. Ward nurses scored participants’ p-ADL function as soon as possible after inclusion (*n* = 36). Two MDs completed APACHE II and CIRS retrospectively based on data at admission. We collected relevant demographic information from patient records.

### Data analysis

To describe the data we used means, standard deviations (SD) and ranges. We used simple (unadjusted) linear regression to explore associations between physical function, age, cognitive status, diagnosis of cognitive impairment, p-ADL-function, comorbidity, deviating vital signs and physical activity. Based on results from the simple linear regression, we created a multiple (adjusted) regression model to test if physical function, age, diagnosis of cognitive impairment, p-ADL-function and comorbidity were significantly associated with participants’ physical activity. We did not include MMSE score in the model because we only had MMSE score for 30 patients. To avoid too many variables in the multiple regression model and because CIRS also provides information about acute illness we did not include APACHE II score in the model. We checked normality of residuals by visual inspection of QQ-plots. We have considered *p*-values under 0.05 as significant and have reported 95% confidence intervals (CI) where relevant. We completed all analysis using SPSS version 22.

## Results

**Fig. 1:**
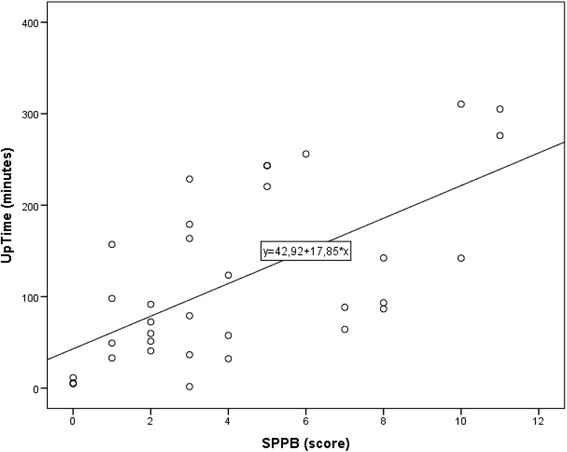
Scatterplot of participants’ SPPB-score (*X-axis*) plotted against time in *upright position* (*Y-axis*) with regression line added

We consecutively included 43 patients. Of these, 38 completed activity monitoring early after hospital admission (mean 3.2 days after admission) and were included in the analyses. The patients with missing activity monitoring data (one out of five were female) were older (88.2 years vs 82.9 years), had different scores on SPPB (3.0 vs 4.3), but had only minor differences in mean MMSE score (19.0 vs 19.3), mean BI score (15.8 vs 16.4) and mean CIRS score (17.0 vs 17.6). Table 1 shows participants’ characteristics. The participants wore the accelerometer on average 5.39 days (SD 3.77). The average participant spent 1 h and 57 min in upright position 1 day early after admission to hospital, but the variation between participants was large (SD 90.1 min). Figure 1 shows upright time during night, morning, afternoon, and evening.

**Table 1:** ᅟ

	N	Mean (SD)	(Range)
Demographic characteristics			
Age (years)	38	82.9 (6.3)	(67.6-92.5)
Length of stay (days)	38	11.1 (7.8)	(3–45)
Female (%)	26 (68.4)		
Home living (%)	37 (97.4)		
Main diagnosis of cognitive impairment (%)	16 (42.1)		
Main diagnosis of gait and balance problems (%)	9 (23.6)		
Died during hospital stay	3 (7.9)		
Physical Activity:			
Upright time per day (min)	38	117.1 (90.1)	(1.7-310.5)
Number of upright events per day	38	42.2 (21.5)	(4–93)
Length of upright events (min)	38	2.5 (1.3)	(0.4-5.7)
Maximum length of upright events (min)	38	12.4 (8.5)	(0.6-28.5)
Upright event variability (IQR^a^, min)	38	2.3 (1.4)	(0.1-5.7)
Mobility:			
Gait speed (m/s)	30	0.6 (0.2)	(0.2-1.3)
SPPB^b^ (0–12)	34	4.3 (3.3)	(0–11)
ADL function, BI^c^ (0–20)	36	16.4 (4.4)	(5–20)
Cognitive function, MMSE^d^ (0–30)	30	19.3 (5.3)	(7–30)
Somatic disease			
CIRS^e^ (0–56)	38	17.0 (4.2)	(8–25)
APACHE^f^ II (0–71)	38	9.3 (3.4)	(6–24)

^a^Inter Quartile Range

^b^Short Physical Performance Battery

^c^Barthel Index

^d^Mini Mental Status Examination

^e^Cumulative Illness Rating Scale

^f^Acute Physiology and Chronic Health Evaluation

**Fig. 2:**
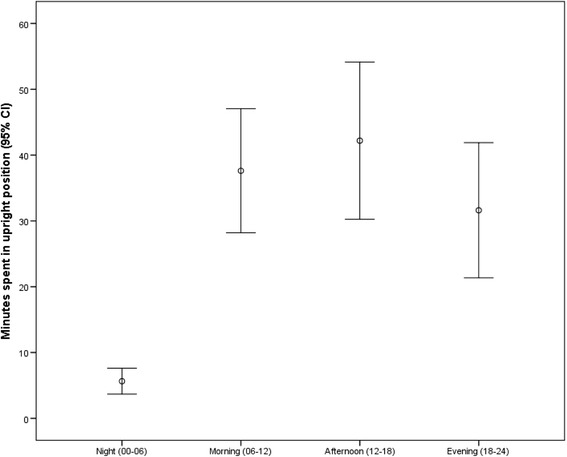
Participants’ *upright time* during night, morning, afternoon, and evening (*n* = 38)

In the unadjusted regression model, increasing BI score and increasing SPPB score were associated with increased level of physical activity, whereas increasing CIRS score was associated with decreasing level of physical activity. In the fully adjusted model only increasing SPPB score was significantly associated with increasing level of physical activity (Table 2). For each one unit increase in SPPB, the expected increase in upright time was 11.74 min. The overall model fit for the fully adjusted model was R^2^ 0.431, with an overall *p*-value 0.001. Figure 2 illustrates the relation between SPPB-score and time in upright position.

**Table 2:** Linear regression with time in upright position (minutes) as dependent variable. Association between SPPB, Age, Diagnosis of cognitive impairment, BI and CIRS and time in upright position. Regression coefficients β, 95% confidence interval (CI) for regression coefficients and *p*-value in the unadjusted and fully adjusted regression model

Independent variable	Unadjusted			Fully adjusted		
	Regression coefficient β			Regression coefficient β		
	Estimate	95% CI	*p*-value	Estimate	95% CI	*p*-value
Mobility (SPPB)^a^	17.85	9.91 to 25.80	<0.001	11.74	0.13 to 23.34	0.048
Age	−2.37	−7.12 to 2.39	0.319	1.07	−3.16 to 5.30	0.608
Diagnosis of cognitive impariment	−1.73	−69.97 to 66.50	0.96	−8.51	−74.83 to 57.80	0.794
ADL-Function (BI)^b^	12.54	6.78 to 18.29	<0.001	6.32	−1.92 to 14.56	0.127
Morbidity (CIRS)^c^	−10.23	−16.65 to - 3.82	.003	−4.41	−11.59 to 2.77	0.218

^a^Short Physical Performance Battery

^b^Barthel Index

^c^Cumulative Illness Rating Scale

## Discussion

In this study of hospitalized older adults, we found that participants’ mean time in upright position 1 day early after admission was almost 2 hours. The variation in upright time was large with a standard deviation of 1.5 h. In an unadjusted regression model there were significant associations with physical activity for both physical function, p-ADL function and morbidity. In the adjusted model participants’ physical function was the only factor significantly associated with their level of physical activity highlighting that physical function is more important than cognitive status, morbidity, age and independence in daily life functions in predicting physical activity during hospitalization.

The level of physical activity, measured as time in upright (standing and walking) position, in our case-mix of patients 82.9 years of age is higher than reported in comparable studies. Callen and colleagues reported median time of ambulating in the hallways at the ward of only 5.5 min per day. They collected data in 1998 in a medical ward in an academic hospital in Wisconsin, US. The participants had a mean age of 74 years [5]. Brown and colleagues collected data in 2006/2007 from a medical ward in Alabama, finding that hospitalized male veterans with mean age 73.9 years spent 55 min per day standing and/or walking [4]. Pedersen and colleagues reported that medical in-patients with mean age 84.5 years spent about 66 min per day standing and/or walking [6], these data were collected in Copenhagen, Denmark in 2010/2011. Ostir and colleagues reported that 224 patients with mean age 76.1 years acutely admitted to an Acute Care for Elders hospital in Texas, US, due to medical conditions were active 80 min the first 24 h in hospital (9). These data were collected in 2008/2009. Villumsen and colleagues reported results from an acute geriatric ward reporting an average of 83 min per day in upright position for patients with mean age 84 years [7]; these data were collected in Aalborg, Denmark in 2012.

Based on results from Ostir [9], Villumsen [7] and the present study, we may speculate if the particular focus on mobilization in geriatric wards is one factor explaining why studies from geriatric wards report higher levels of physical activity than studies on patients in general medical wards [4–6]. Our group has previously compared the level of physical activity among hip fracture patients in a geriatric ward and in an orthopedic ward [10], finding a higher level of physical activity for patients in the geriatric ward. One factor discussed in this paper was the continuous focus on mobilization in the ward, where physiotherapists have been an integrated part of the ward staff for many years and where nurses focus on mobilization and cooperate closely with the physiotherapists if a patient is hard to mobilize. We believe this focus on mobilization and physical activity through many years has resulted in an intensive and well-working mobilization regime that to some extent explains the results also in this study.

Our study population illustrates that geriatric patients are heterogeneous in terms of cognition, physical function, p-ADL function, level of morbidity and level of physical activity. Consequently, the staff must assess all patients on an individual basis to make individualized mobilization regimes. Some will be able to maintain an acceptable level of physical activity alone, but others need close follow up by the staff, implicating that sufficient number of nurses and physiotherapists are key factors to achieve successful mobilization. Recognition of such aspects can also have contributed to the higher level of physical activity in our ward.

There might as well be methodological explanations to the differences in level of physical activity. Our data are for most patients collected on day three, while some studies [4, 6, 8] report data from the entire period of hospitalization and thereby include the first 2 days where the patients probably are less physically active due to acute illness. Because the observational study by Callen [5] only includes observations in the hallways at daytime this study probably underestimates the total amount of physical activity. This study is almost 20 years old, and the focus on mobilization in general has increased during these years. On the other hand, our patients are older and more cognitively impaired than the patients in the two oldest studies [4, 5], and diagnoses of falls and gait disturbances were frequent.

In our study, physical function as measured with SPPB early after admittance was the only variable associated with level of physical activity. Unexpectedly, there was no association between age, diagnosis of cognitive impairment, BI score, CIRS score and physical activity. The association between physical function and physical activity in hospital settings is important to highlight, because patients with physical impairment are more difficult to mobilize, and run a higher risk for further loss of function. Our results implicate that there should be an increased, individualized and specific focus on mobilization in the group of patients with poor physical function.

The main limitations of this study are the small sample size and the lack of complete data for many important variables. It is also worth noting that this case-mix lack severe organ failure and deviating vital signs and that few participants had diagnoses of acute medical illnesses like severe infections, myocardial infarction, heart failure or stroke. Our results are therefore not applicable to wards taking care of elderly patients with immediate life-threatening acute illnesses. The major strengths are the objective measurement of activity by activPAL and the inclusion of patients with cognitive and functional impairment. The fact that this is an observational study and not an intervention study with extra focus on physical activity, is another strength increasing the external validity. On the other hand, there is an opportunity that both patients and staff, being aware of the accelerometer, have walked more and promoted activity more and thereby induced a bias leading to overestimation of the level of physical activity.

## Conclusions

In this study, we document a relatively high level of physical activity among hospitalized geriatric patients, where the mean time spent in an upright (standing and walking) position 1 day early after admission was close to 2 hours. In this sample, physical function measured by SPPB was the only factor significantly associated with physical activity, while there was no association between age, cognitive impairment, p-ADL-function, burden of comorbidity and physical activity. Our study indicates that it is possible to mobilize even acutely admitted geriatric patients and that SPPB could be a useful screening tool for physical activity. Further research is necessary to evaluate if mobilization regimes based on such simple screening tools could improve outcomes in geriatric patients.

## Abbreviations

APACHE: Acute Physiology and Chronic Health Evaluation
BI: Barthel index
CIRS: Cumulative illness rating scale
MD: Medical doctor
MMSE: Mini mental status examination
p-ADL: personal Activity of Daily Living
SD: Standard deviation
SPPB: Short Physical Performance Battery
